# Refractory trigeminal neuralgia treatment outcomes following CyberKnife radiosurgery

**DOI:** 10.1186/s13014-014-0257-8

**Published:** 2014-12-14

**Authors:** Sana D Karam, Alexander Tai, James W Snider, Shilpa Bhatia, Edward J Bedrick, Abdul Rashid, Ann Jay, Christopher Kalhorn, Nathan Nair, K William Harter, Sean P Collins, Walter Jean

**Affiliations:** Department of Radiation Oncology, University of Colorado Hospital, Aurora, CO USA; Department of Radiation Oncology, Medstar Georgetown University Hospital, Washington, DC USA; Department of Radiation Oncology, University of Maryland, Baltimore, MD USA; Department of Radiation Oncology, Georgetown University Hospital, 3800 Reservoir Road, NW, Washington, DC 20007 USA; Department of Neurosurgery, Georgetown University Hospital, Washington, DC USA; Department of Biostatistics and Bioinformatics, University of Colorado, Aurora, CO USA

**Keywords:** Trigeminal Neuralgia, Tic doloreux, Radiosurgery, Cyberknife, Long term

## Abstract

**Introduction:**

A handful of studies have reported outcomes with CyberKnife radiosurgery (CKRS) for the treatment of trigeminal neuralgia. However, the follow-up has been short with no minimum follow-up required and have included patients with short duration of symptoms. Here we report our institutional experience on patients with a minimum follow-up of 1 year and a median follow-up of 28 months (mean 38.84 months).

**Methods:**

Twenty-five patients with medically and surgically intractable TN received CKRS with a mean marginal radiation dose of 64 Gy applied to an average isodose line of 86% of the affected trigeminal nerve. Follow-up data were obtained by clinical examination and telephone questionnaire. Outcome results were categorized based on the Barrow Neurological Institute (BNI) pain scale with BNI I-III considered to be good outcomes and BNI IV-V considered as treatment failure. BNI facial numbness score was used to assess treatment complications.

**Results:**

A large proportion of patients (42.9%) reported pain relief within 1 month following CKRS treatment. The mean time to recurrence of severe pain was 27.8 months (range 1–129 months). At median follow-up of 28 months (mean 38.84 months), actuarial rate of freedom from severe pain (BNI ≥ III) was 72%. At last follow-up 2 (8%) patients had freedom from any pain and no medications (BNI I) and the majority (48%) had some pain that was adequately controlled with medications. Seven patients (28%) had no response to treatment and continued to suffer from severe pain (BNI IV or V). Patient’s diabetic status and overall post-treatment BNI facial numbness scores were statistically significant predictors of treatment outcomes.

**Conclusion:**

CKRS represents an acceptable salvage option for with medically and/or surgically refractory patients. Even patients with severely debilitating symptoms may experience significant and sustained pain relief after CKRS. Particularly, CKRS remains an attractive option in patients who are not good surgical candidates or possibly even failed surgical therapy. This data should help in setting realistic expectations for weighing the various available treatment options.

## Introduction

Trigeminal neuralgia (TN) is a debilitating condition characterized by agonizing, paroxysmal, and lancinating pain [1]. Although the incidence of TN was thought to be less than 5 per 100,000 patient-years based on epidemiologic data from Olmstead County, Minnesota [2] more recent studies have found TN to be much more common with incidence rates ranging from 12.6 to 28.9 per 100,000 patient-years [3]. Most patients suffering from trigeminal neuralgia (TN) respond to medical or surgical treatment, nonresponders have limited options [4]. Second-line treatment modalities are utilized in patients whose symptoms are intractable or who cannot tolerate medication. These include surgical procedures such as microvascular decompression (MVD), and ablative procedures such as percutaneous balloon microcompression, radiofrequency rhizotomy, glycerol rhizolysis, and radiosurgery. While GammaKnife Radiosurgery has been shown to be effective in obtaining long term pain relief in patients afflicted with this disease, there have been only a handful of reports with the CyberKnife Radiosurgery (CKRS) system (Accuray, Inc., Sunnyvale, CA) [5-7]. The ease of administration and non-invasive nature of this non-isocentric treatment modality makes it an appealing procedure for patients and treating radiosurgeons. Here we present the longest institutional outcomes reported to date for CKRS in the treatment of TN. Our analysis also represents the only one in which the inclusion criteria are limited to patients with a pretreatment BNI of IV or V and with a minimum follow-up of 12 months.

## Materials and methods

### Patient characteristics

After institutional review board (IRB) approval by Georgetown University Hospital IRB, patient demographic characteristics, clinical presentation, treatment history, and the radiosurgical modality were retrospectively reviewed.

Patients were also followed-up by a telephone questionnaire that was conducted by a medical personnel who were not involved in treatment. Patients were questioned about the time to the onset of pain relief, the degree of pain relief and treatment complications. Based on the Barrow Neurological Institute (BNI) score for TN, we classified pain relief after treatment into five grades. A BNI I score corresponded to complete pain relief without medications; BNI II score, some pain but not requiring medications; BNI III score, some pain but adequately controlled with medications; BNI IV score, some pain not adequately controlled with medication; and BNI V score, severe pain or no pain relief. The BNI facial numbness score was used to assess complications. A BNI I score corresponded to no facial numbness; BNI II score, mild facial numbness, not bothersome; BNI III, facial numbness somewhat bothersome; and BNI IV score, facial numbness, very bothersome.

Between July 2002 and February 2013, 30 patients with severe refractory TN and with minimum follow-up of 12 months underwent CK at our clinic. Five patients had no follow up information and could not be contacted by phone so they were excluded for a final sample size of 25 patients. Indications for CK included intractable pain, with a pretreatment BNI score or IV or V, refractory to standard medications and failure of previous invasive procedures. The median follow-up was 28 months (range 12–129 months; mean 38.84 months) and a summary of patient characteristics is provided in Table 1.

**Table 1:** **Clinical demographic characteristics in 36 patients with medically intractable trigeminal neuralgia treated with gamma knife radiosurgery (GKRS)**

**Characteristic**	**Value**
**Gender**	
Male	13 (52.0%)
Female	12 (48.0%)
**Mean age (range)**	65 (43–86)
**Prior surgery**	20 (80.0%)
**Median duration in years (range)**	8.5 (4–28)
**Pain distribution**	
V1	3 (12.0%)
V2	2 (12.0%)
V1,2	3 (12.0%)
V3	1 (4.0%)
V2,3	12 (48.0%)
V1,2,3	3 (12.0%)
**Side of pain**	
Right	11 (44.0%)
Left	14 (56.0%)
**Multiple sclerosis**	4 (16.0%)

### Radiosurgery technique and dosimetry

The CKRS system (Accuray, Inc., Sunnyvale, CA, USA) uses a 6-MV X-band linear accelerator (LA) mounted on a fully articulated robotic arm. During treatment, two orthogonally positioned x-ray detectors provide real-time imaging of bony anatomy allowing for intrafraction movement correction. Treatment was generally administered on an outpatient basis with each treatment lasting ∼ 45–90 min.

**Figure 1:**
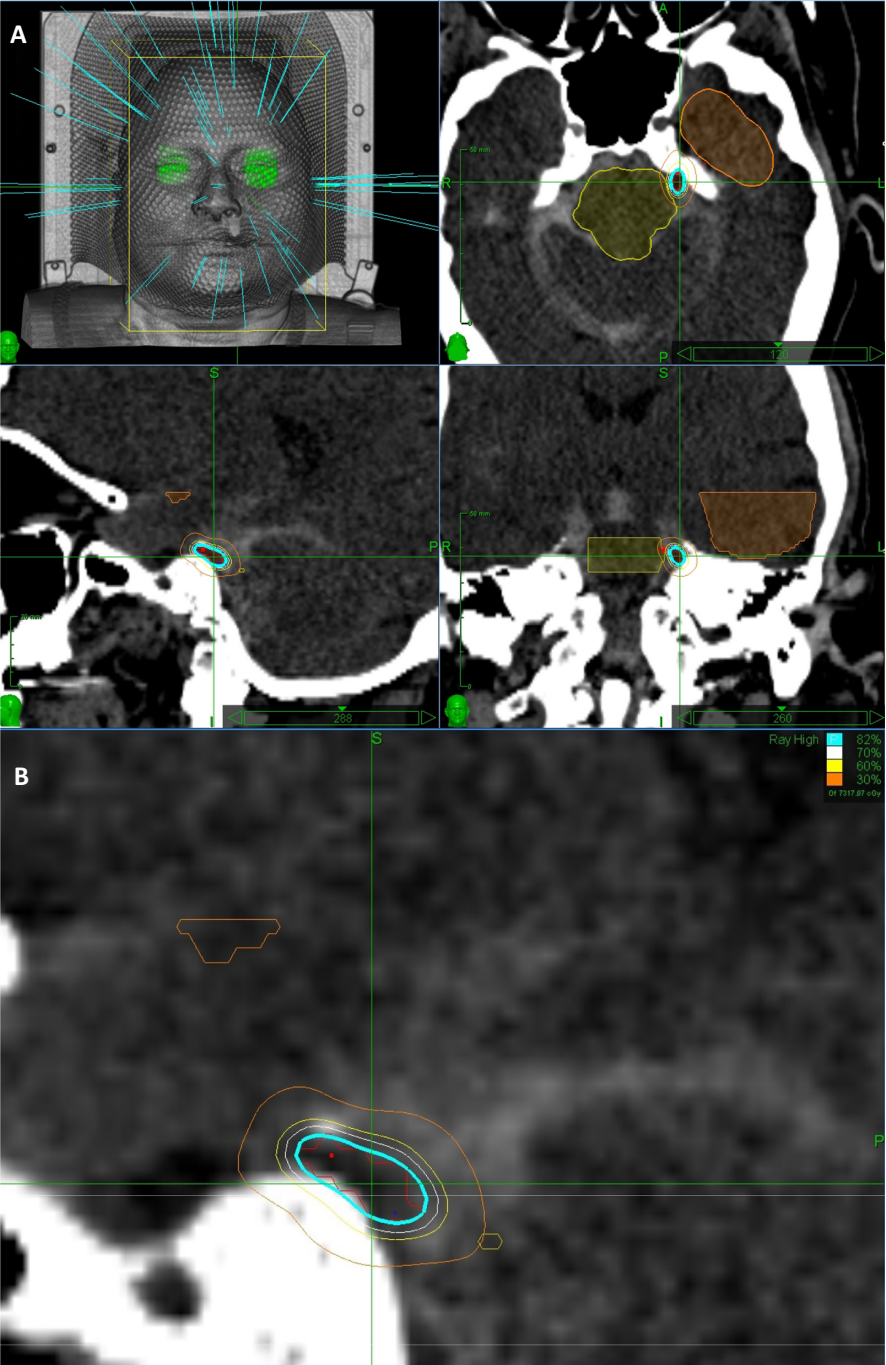
**Example of CyberKnife radiosurgery plan for trigemial neuralgia. A**. Screen shot taken from the CyberKnife (Accuray, Inc., Sunnyvale, CA) treatment planning workstation depicting a representative radiosurgical plan for trigeminal neuralgia. Yellow and brown contours refer to the brainstem and temporal lobe, respectively. **B**. Higher magnification image illustrating the dose distribution within the target volume.

Patients were immobilized in the supine position with an Aquaplast facemask (WRF/Aquaplast Corp., Wyckoff, NJ, USA). All patients underwent an iopamidol-enhanced CT cisternography with 1.25-mm contiguous slices was used to visualize the segment of the trigeminal nerve in the prepontine cistern. A lumbar puncture was performed to inject 10 mL to 12 mL of contrast material. The trigeminal nerve was readily identified on the planning workstation and a segment of the nerve was marked as the target (mean volume range, 25–71 mm3). The target included the cisternal segment of the trigeminal nerve extending to the gasserian ganglion. The radiation oncologist, neurosurgeon, and radiation physicist performed tumor delineation, dose selection, and planning. Inverse planning was used to determine the dose to the target volume while minimizing the dose to normal tissue. A mean marginal prescription dose of 64.12 Gy (range, 60-80Gy) was used over the course of this series. The average prescription isodose line was 86%, whereas the dose at the edge of the brainstem was kept to less than 30% of isodose line touching the brain stem, which gives about 22.5 Gy to the brain stem. Figure 1 shows a composite of the treatment. Figure 1 depicts a representative radiosurgical plan for trigeminal neuralgia.

### Statistical analysis

Treatment outcomes were assessed by patient self-reports of pain control and medication usage at last followup. A pain-free outcome was defined as BNI pain score I and pain relief or good outcome was regarded as maintaining a BNI pain score III or better without requiring further surgery. Treatment failure was defined as pain returning to a BNI level of IV or V, or the patient undergoing an invasive surgical procedure due to uncontrolled pain. A recurrence was defined as a relapse to a previous lower level after attainment of any higher level of pain relief. Patients reported the time interval for a response and pain recurrence after CK. The date of treatment failure was considered to be the date at which pain relief became a BNI IV or V score.

Time to BNI class IV to V pain relapse was calculated with the Kaplan-Meier method. Log-rank tests were performed to determine statistical differences between pain relapse curves. We conducted a univariate analysis of several factors hypothesized to influence or predict successful treatment, using Cox regression analysis: age, gender, side of pain, duration of symptoms, prior surgery, diabetic status at diagnosis, pretreatment facial numbness, and new facial numbness. A *p* value <0.05 was accepted as statistically significant. All statistical calculations were performed using SPSS software, version 13.0 (SPSS, Inc., Chicago, IL, USA).

## Results

### Pain relief after CKRS

A large proportion of patients (42.9%) reported pain relief within 1 month following CKRS treatment. Nineteen percent of patients reported relief within 6 months of treatment and another 19% reported relief no relief of symptoms. In 14.3% relief was experienced within 3 months from treatment. The median time to recurrence of severe pain was 19 months (mean 27.8 months; range 1–129 months).

**Figure 2:**
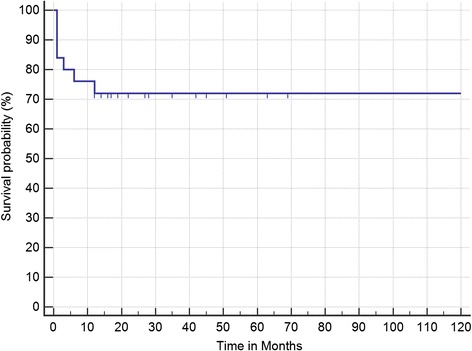
**Kaplan-Meier curve for time from CyberKnife radiosurgery (CKRS) to Barrow Neurologic Institute (BNI) class IV to V pain relapse (i.e., freedom from severe pain).**

Results of Kaplan–Meier analysis of response time after CKRS are displayed in Figure 2. At median follow-up of 28 months (mean 38.8 months), actuarial rate of freedom from severe pain (BNI ≥ III) is 72% (Figure 2). Actuarial median time to return of severe pain was not reached. The patient responses at last follow-up, as determined using the BNI pain intensity scoring system, are listed in Table 2. At last follow-up 2 (8%) patients had freedom from any pain and no medications (BNI I) and the majority (48%) had some pain that was adequately controlled with medications. Seven patients (28%) had no response to treatment and continued to suffer from severe pain (BNI IV or V).

**Table 2:** **Pain response (top 5 rows) and development of facial numbness post treatment (bottom 3 rows) at last follow-up**

**BNI pain intensity scale**	**Number of patients (%)**	
BNI I	2 (8)	
BNI II	4 (16)	
BNI III	12 (48)	
BNI IV	3 (12)	
BNI V	4 (16)	
**BNI facial numbness score**	**Number of patients pretreatment (%)**	**Number of patients posttreatment (%)**
BNI I-II	20 (80)	18 (72)
BNI III	2 (8)	5 (20)
BNI IV	3 (12)	2 (8)

### Treatment related complications

**Figure 3:**
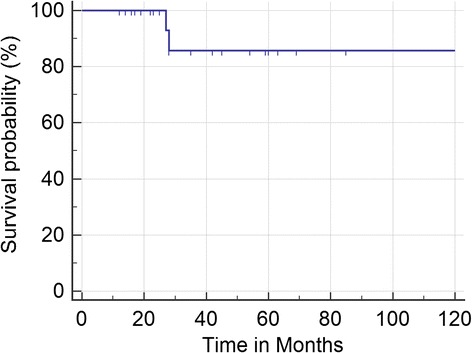
**Kaplan-Meier curve for time from CyberKnife radiosurgery (CKRS) to improvement in Barrow Neurologic Institute (BNI) numbness score.**

The majority of the patients (18 or 72%) did not experience any new bothersome post-treatment facial numbness (Table 2, bottom row). Two patients developed new somewhat bothersome facial numbness (BNI III) and no patients developed any new very bothersome facial numbness (Table 2, bottom row). Indeed, two patients reported improvement in her facial pain from very bothersome facial numbness to no facial numbness (BNI I). Kaplan Meier analysis shows an actuarial rate of improvement of facial numbness of 83% at a median follow-up of 28 months (mean of 38.4 months) (Figure 3). There were no reports of decreased corneal sensation (dry eye syndrome).

### Prognosticators

Univariate analysis for previously published prognosticators of treatment failure is shown in Table 3. Patient’s diabetic status is shown to be a negative prognostic indicator of good outcome (p = 0.05; Table 3). Additionally, a statistically significant correlation is found between overall status of post-treatment BNI facial numbness score and good outcome (Table 3). Patients with an overall bothersome post-treatment BNI facial numbness score (BNI scale III-IV) had improved treatment outcomes (Table 3). There were no other statistically significant prognosticators of outcome (Table 3).

**Table 3:** **Summary of various prognosticators and development of treatment failure (BNI IV-V)**

**Variable**	**Good outcome**	**Treatment failure**	**P-value**
No of patients	18 (72%)	7 (28%)	0.05
Gender			
Male	10	3	0.51
Female	8	4	
Age			
≥70 years	7	3	0.81
<70 years	11	4	
Diabetes			0.05
Yes	2	3	
No	16	4	
Multiple Sclerosis			0.88
Yes	3	1	
No	14	6	
Side of Pain			
Right	9	5	0.38
Left	9	2	
Duration of			
Symptoms			
>8 years	12	4	0.74
<8 years	6	3	
Dose			
60 Gy	14	5	0.68
>60 Gy	4	2	
New Bothersome			0.20
Facial Numbness	4	7	
Yes	14	0	
No			
Post-treatment BNI			
Facial Numbness			
I-II	10	7	0.04
III-IV	8	0	
Prior Surgery			
Yes	13	4	0.40
No	5	3	

## Discussion

Prior studies have reported favorable treatment response rates for CKRS treatment of medically intractable TN with relatively rapid response rates on the scale of a few months and in some studies a few weeks [5-10]. Follow-up in these studies though have been variable between 11 months to over 3 years. This has lead to discrepancies in reported long-term treatment outcomes as several of these studies have observed that the pain relief experienced after CKRS declines over time. Additionally, prognostic factors associated with treatment response have yet to be consistently identified.

In the present study, we report our institutional data on pain outcomes and side effects from CKRS for the treatment of TN with a minimum follow-up of 12 months. In our series, 81% of patients responded to treatment with improvement in TN pain to non-severe levels (BNI III or less), which is consistent with previous studies reporting rates between 67% [10] and 92% [7]. However, our series has been the only one to limit the patient population to patients with severe pain (BNI IV or V) and comprise mostly of patients with surgically intractable TN (80%). Previous studies have observed that patients with surgically intractable TN and those with severe pain undergoing CKRS tend to have significantly worse outcomes than those who are surgically naïve do [5]. Unexpectedly though, prior surgery for TN was not a negative prognostic factor for treatment success in our series. Thus, it is unclear whether the characteristics of our patient series may explain why we did not achieve as high as response rates as some other studies examining CKRS for TN.

Of the patients who responded, the majority of our patients noted improvement in symptoms within 1 month and all reported improvement within 6 months. Villavicencio et al. reported a mean latency to response of 2 weeks in a series of 95 patients [10] and a smaller study reported a mean latency to response of 2.4 days in a series of 13 patients [7]. Other groups, however, have reported mean latencies to response more consistent with our findings (1.92 months and 5.2 weeks in [5,9], respectively). The differences in our results with some of these studies may reflect our higher incidence of surgically intractable TN and our inclusion of patients who only had severe pain (BNI IV or V) pretreatment. Nonetheless, the short duration of response latency period between this study and others emphasizes the importance of discussing further treatment options in patients who have not achieved pain relief within 6 months.

Additionally, our long-term results also are consistent with other studies reporting long-term data. Our actuarial freedom from severe pain rate was 72% at 3.2 years. Other studies have reported actuarial or observed rates of sustained pain relief between 50 to 80% at around 3 years follow-up [9,10]. Thus, our results add further weight to the findings of other studies that CKRS for TN has the potential for sustained significant pain relief. In contrast to other studies though, the mean time to recurrence of severe pain in our patients was 27.8 months, which appears to be much longer than those of other studies reporting recurrence times between 9 and 18 months. It is unclear as to why our mean time to recurrence of severe pain was such longer than other reported rates. Possibly, differences in our patient population (i.e. higher proportion of surgically intractable patients, inclusive of only patients with BNI IV and V pain, younger patients) may explain this discrepancy.

A common complication associated with CKRS for TN, which was experienced by a few patients in this series, is the development of facial numbness. Previous studies have reported variable rates of bothersome facial numbness post-treatment ranging from no patients [6] to over 40% of the patients [9]. A consistent observation is that higher maximal and marginal doses are associated with higher incidence of facial numbness, though they are also associated with increased treatment response and sustained pain relief as well [11]. We observed this in our series as well. The amount of radiation delivered to the brainstem also is known to play a large role in the development of post-treatment facial numbness. In our study, we limited brainstem radiation to 22.5 Gy and our average marginal dose was 64.12 Gy. At last follow-up, only 2 patients reported the development of or worsening of facial numbness to bothersome levels. This rate is consistent with previous studies utilizing treatment doses similar to ours (Adler et al. [5] – 15% BNI III or greater with marginal of 58.3, Villavencencio et al., 2007 – 28% BNI III or greater with marginal of 60 [10]). Fariselli et al. [6] who reported that no patient experienced the development of bothersome facial numbness limited their brainstem radiation dose to 14 Gy. Thus, limiting the radiation dose to the brainstem and the marginal dose likely would have reduced the rate of developing facial numbness post-treatment in our series. However, it likely would have also reduced the treatment response.

An interesting finding in our study was that diabetic patients had significantly worse pain outcomes after CKRS for TN than patients who did not have a history of diabetes. In our series, 3 of the 5 diabetic patients experienced treatment failure (BNI of IV or V at last follow-up), whereas, only 4 of 20 non-diabetic patients experienced treatment failure. Another study examining factors associated with the durability of pain relief after CKRS for TN also reported that diabetes appeared to be a negative prognostic indicator [11]. The exact mechanism by which diabetic status affects pain outcomes after CKRS for TN pain is unknown. Though one may speculate that the impaired healing capability of diabetics or pre-existing neuropathy may contribute to worse outcomes or more severe disease, further studies examining this relationship are warranted. Other prognostic factors examined other than diabetes and facial numbness such as multiple sclerosis, age, gender, laterality of pain, duration of symptoms or radiation dose did not appear to be prognostic of pain outcome.

Lastly, SRS has long been reserved for TN patients who are not surgical candidates or have comorbidities conferring them a shorter life expectancy. However, a recent study suggested that CKRS might be preferable to surgical therapy, in particular MVD, in certain circumstances due to its economic benefits. Tarricone et al. reported that CKRS proved to be more cost-effective than MVD yet possessed equivalent clinical effectiveness as MVD did [12]. These authors’ analyses revealed a cost difference of 2,250 Euros, which they attributed mostly to the cost of the surgical procedure and the cost of the hospital stay for an MVD. Long-term pain control past 6 months though was not included in their analysis and CKRS has yet to demonstrate in any series the same sustained clinical effectiveness as MVD over several years. Nevertheless, given the successful long-term results obtained in our series, CKRS appears to be an attractive option that is clinically effective, sustained and economic.

There are several limitations to this study. The small sample size and retrospective nature of this study design limit the power of our outcome observations. Additionally, although pain intensity and numbness scales are validated tools for the quantification of pain and numbness, they are subjective outcome measures because they are dependent on personal interpretations and variation. The results of this study should, however, help clinicians provide important information to patients so they can have realistic expectations and be able to weigh the risks and benefits relative to the various available treatment options.

## Conclusions

CKRS is a safe, effective, minimally invasive and potentially cost-effective treatment modality for patients with medically intractable TN or those who are ineligible or refuse open surgery. Our results demonstrate that a CKRS treatment is associated with good outcomes in the majority of patients with sustained relief of TN pain in most responding to therapy. Our observations also further support the relationship between the development of facial numbness and treatment success and suggest that diabetic status might be a negative prognostic factor in response to CKRS for TN.
